# Contrast-enhanced ultrasound of small cell carcinoma in urinary bladder: a case report and review of literature

**DOI:** 10.1186/s12885-017-3692-8

**Published:** 2017-11-10

**Authors:** Meixiang Zhang, Chengcheng Niu, Ming Zhang, Qinghai Peng, Minzhi Ouyang

**Affiliations:** Department of Ultrasound Diagnosis, The Second Xiangya Hospital, Central South University, Changsha, Hunan 410011 China

**Keywords:** Small cell carcinoma of the bladder, Contrast-enhanced ultrasound, Conventional ultrasound

## Abstract

**Background:**

Small cell carcinoma of the urinary bladder (SCCB) is a relatively rare malignant bladder tumor, and few reports have investigated the microvasculature of SCCB imaged using contrast-enhanced ultrasound (CEUS).

**Case presentation:**

A 63-year-old female was admitted to our hospital after experiencing painless gross hematuria for one week. The gray-scale ultrasound (US) demonstrated a 4.8 × 3.4 × 3.6-cm^3^ hypoechoic mass in the apex of the urinary bladder with a wide base and an irregular surface; the mass did not move with changes in body position. Color Doppler flow imaging (CDFI) showed rich blood flow in the mass. CEUS with low mechanical index (MI) of 0.06 confirmed a highly enhanced 5.0 × 3.3 × 3.8 cm^3^ mass within the bladder at the apex wall. The time-intensity curves (TICs) showed a wash-in time of 10 s, a time to peak (TTP) of 33 s, a signal intensity (SI) of 62.7% and a wash-out time > 60 s. Finally, the transurethral resection of the bladder tumor (TURBT) was performed, and the pathological examination proved the diagnosis of SCCB.

**Conclusion:**

CEUS can provide valuable information related to the rich microvasculature of SCCB, which may be helpful in its diagnosis.

## Background

Small cell carcinoma of the urinary bladder (SCCB) is a relatively rare malignant bladder tumor with a reported proportion of 0.5% to 1% of primary bladder cancers [1–5]. Owing to its more aggressive nature and poorer prognosis than primary urothelial carcinoma of the bladder, SCCB is mostly identified and diagnosed at an advanced stage, with tumor metastasis detected in more than 60% of reported SCCB patients [6–8].

Contrast-enhanced ultrasound (CEUS) involves the application of ultrasound contrast agents (UCAs), microbubbles with a diameter similar to red cells, to obtain enhanced imaging of the parenchymal microvasculature of organs and tissues on the basis of conventional sonography. Conventional sonography is the most frequent approach used to detect bladder lesions, but its diagnostic specificity is relatively low, for it may be difficult to differentiate them from other benign lesions such as blood clots according to ultrasonographic features only. Some authors have demonstrated that CEUS is better than conventional ultrasound (US) in assessing bladder tumor grade based on the clear imaging of muscle infiltration [9]. According to Nicolau et al., CEUS exhibited an extremely high sensitivity for the presence of bladder cancer per patient (90.9%); the sensitivity for the number of detected bladder tumors was 65.5%, due to the high number of <5 mm detected by cystoscopy [10]. Drudi et al. demonstrated that CEUS has potential in bladder tumor grading using the pattern of time-intensity curves (TICs) in most cases [11, 12]. Guo et al. found TICs of CEUS reflect the tumor microvessel density in bladder urothelial carcinoma and may be helpful for evaluating tumor angionesis [13]. However, few studies have reported the use of ultrasonography for SCCB. To our knowledge, the case described here is the first case in which CEUS is used in the diagnosis of SCCB.

## Case presentation

**Fig. 1:**
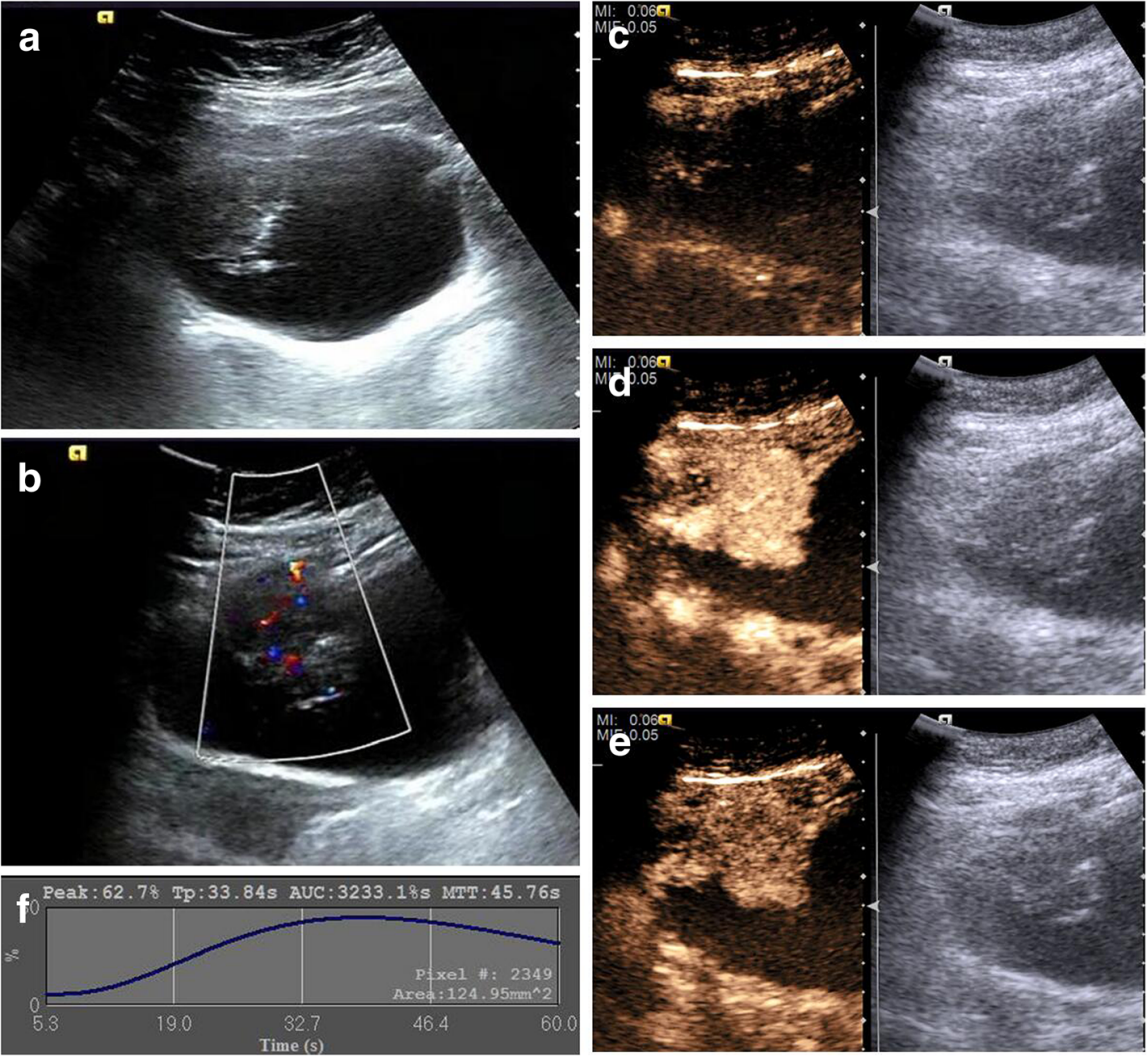
US images of small cell carcinoma of the bladder. **a**. Abdominal sonography revealed a hypoechoic mass in the apex of the urinary bladder. **b**. CDFI showed a rich blood flow signal inside this mass. **c**. CEUS imaging showed the mass began to undergo enhancement (wash-in time) from its periphery to its center at 10 s. **d**. CEUS imaging showed persistent high peak enhancement of the mass at 40 s. **e**. CEUS showed the enhancement signal of the mass was equal to the bladder wall at 120 s. **f**. Time-intensity curve showed wash-in time of 10 s, TTP of 33 s, SI of 62.7% and wash-out time > 60 s

A 63-year-old female was admitted to our hospital after experiencing painless gross hematuria for one week. Abdominal US (Siemens Acuson S3000, Mountain View, CA, USA)with a 6C1 HD probe, probe frequency ranging from 3.0 to 5.5 MHz, revealed a solitary 4.8 × 3.4 × 3.6 cm^3^ hypoechoic mass in the apex of the urinary bladder (Fig. 1a). The mass exhibited a wide base and an irregular surface, and it did not move with changes in body position. Color Doppler flow imaging (CDFI) showed rich blood flow signals in the mass (Fig. 1b). CEUS was performed using Cadence contrast pulse sequencing technology (CPS, mechanical index (MI) = 0.06) with a bolus intravenous injection of 1.5 ml of SonoVue (Bracco, Milan, Italy), followed by 5 ml of a concurrent saline flush when the timing started. The dynamic imaging of CEUS of the mass was recorded by the machine, and the video was reviewed and TIC was extracted from the region of interest (ROI) in the lesion subsequently after the procedures were finished. The CEUS analysis was performed with dedicated software (Contrast Dynamics, USA), and the TIC within selected ROI was acquired. The ROI was marked as a polygon on the lesion to be studied. The mass began to undergo rapid high enhancement from the periphery to the center at 10 s (wash-in time) (Fig. 1c). At 33 s, the enhancement of the mass peaked (time to peak, TTP), and high enhancement was continuously maintained until 40 s (Fig. 1d). Then microbubbles in the mass began to wash out, and the enhancement decreased to a level equal to that of the bladder wall at 82 s (Fig. 1e). The microbubbles in the mass completely washed out after 300 s. The size of the enhanced mass was calculated as 5.0 × 3.3 × 3.8 cm^3^. Based on our experience and the rich microvasculature revealed by CEUS imaging, we inferred that the mass was likely a malignant tumor.

**Fig. 2:**
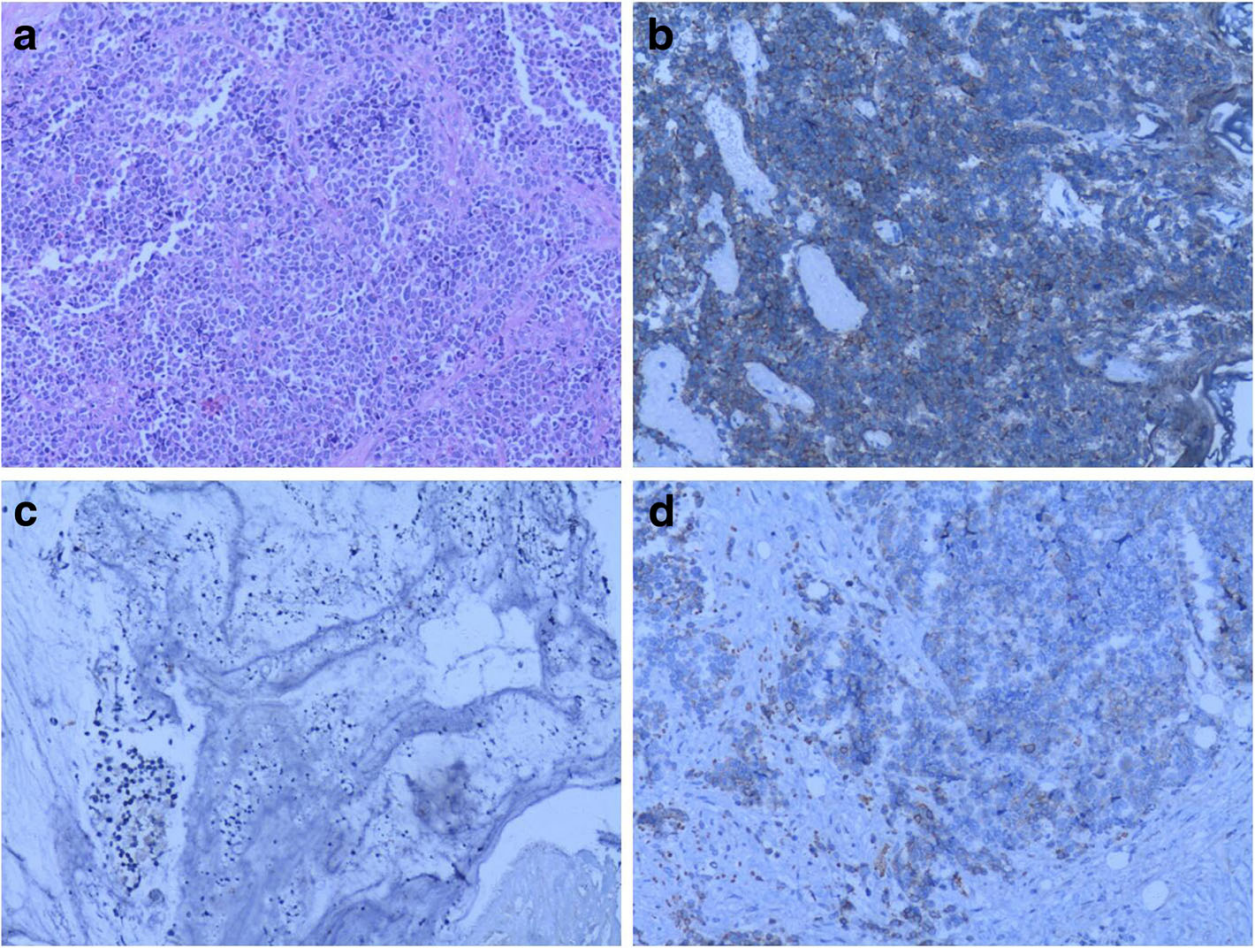
Histopathology of small cell carcinoma of the bladder. **a**. Hematoxylin and eosin (H&E) staining of the biopsy specimen revealed nests of small cells with scant cytoplasm and abundant nuclei. b-d. Immunostaining of the biopsy specimen indicated that tumor cells were positive for Syn (**b**), NSE (**c**), and CD56 (**d**). (magnification, ×200)

Bladder endoscopy revealed a large polypoid tumor in the apex of the urinary bladder, and the transurethral resection of the bladder tumor (TRUBT) was undertaken. Histopathological examination showed that the tumor had invaded the lamina propria and deep muscularis of the bladder wall (Fig. 2a). Immunohistochemically, tumor cells were positive for neuron-specific enolase (NSE, Fig. 2b), synaptophysin (Syn, Fig. 2c), cytokine 56 (CD56, Fig. 2d), cytokine 99 (CD99), P63 (++), P16, P53 (90%+), and Ki-67 (90%+). Based on these findings, the tumor was diagnosed as high grade SCCB.

## Discussion and conclusions

Bladder cancer is the most common malignant tumor in the urinary system. Primary urothelial carcinoma accounts for more than 90% of primary bladder carcinomas [12]. SCCB is a rare malignant tumor of the urinary system with an incidence of 0.5% to 1% of primary bladder cancers. Both of these malignancies have similar clinical manifestations and imaging characteristics. However, compared with primary urothelial carcinoma, SCCB is more aggressive and has a poorer prognosis. As a result, most SCCB patients are in advanced stages of their disease when admitted to the hospital. Several studies have shown that the combined 5-year survival rate for all stages of SCCB is 19% [6].

Computed tomography (CT), magnetic resonance image (MRI) and US have been extensively used for the identification and staging of bladder lesions in clinical practice. CT and MRI have obvious advantages in the detection and staging of tumors, especially for the assessment of deeply infiltrating tumors and lymph nodes metastasis. However, CT cannot show individual layers of the bladder wall; therefore, it is not reliable for estimating the degree of tumor invasion. MRI is contraindicated in some patients with metal implants and the potential exists for overstaging bladder cancer because of hyperemia and acute edema [14].

A few studies have explored the application of CEUS in the diagnosis of bladder tumors, in spite of its usage in SCCB being scarce. Drudi et al. found that TICs of high grade bladder tumor showed slow wash-in, with a high maximum signal intensity (SI) and fast wash-out, while TICs of low grade bladder tumors showed faster wash-in, lower SI and slower wash-out in 2012 [11]. Subsequently, they detected different grading urothelial cell carcinoma (UCC) with the same wash-in time of 13 s, with low grade UCC showing TTP <28 s, SI <45% and wash-out time of 40 s and high grade UCC showing TTP >28 s, SI >50% and wash-out time of 58 s in 2014 [12]. Gupta et al. defined two types of CEUS TIC for different graded UCC. Type A curve is defined by rapid and high peak enhancement and fast wash-out time, correlating well with high grade UCC. Type B is defined by an early enhancement peak but slow plateau and very slow wash-out time, correlating well with low grade UCC [15]. In our study, CEUS with TIC of SCCB showed wash-in time of 10 s, TTP of 33 s, SI of 62.7% and wash-out time > 60 s. This is similar to the results of high grade UCC with high SI and slow wash-out time as reported by Drudi et al. in 2014 [12]. According to Guo et al., with increasing malignancy in bladder cancer, angiogenesis as a result of greater arteriovenous fistulas formation, tortuous blood vessels, and aggravation of interstitial edema were related to the wash-out of contrast agent being slowed down in blood vessels [13]. In agreement with the aggressive biological behavior of SCCB, the TIC parameter of SCCB in our study showed high peak enhancement and a very slow wash-out time, consistent with the high-grade bladder cancer [13].

However, CEUS has some limitations. It is difficult to use CEUS to identify small lesions less than 5 mm. According to Nicolau et al., the sensitivity of CEUS for detecting bladder cancer was extremely high for tumors larger than 5 mm (94.7%) but extremely low for tumors smaller than 5 mm (20%); CEUS also exhibited an extremely low negative predictive value (28.57%) for tumors smaller than 5 mm [10]. Moreover, compared with the other medical imaging technologies, CEUS is more reliant on the practice and experience of the physician.

In conclusion, we present the CEUS features of a case of SCCB. Our findings indicate the TIC parameters of SCCB are consistent with the enhancing patterns of high grade bladder cancer; however, whether they are a typical presentation of SCCB and whether they can be used as its diagnostic index depends on further research based on an analysis of more samples. Nevertheless, histopathological examination remains the gold standard for diagnosing this disease.

## Abbreviations

CD: cytokine
CDFI: color Doppler flow imaging
CEUS: contrast-enhanced ultrasound
CPS: contrast pulse sequencing
CT: computed tomography
MI: mechanical index
MRI: magnetic resonance image
NSE: neuron-specific enolase
ROI: region of interest
SCCB: small cell carcinoma of the urinary bladder
SI: signal intensity
Syn: synaptophysin
TICs: time-intensity curves
TTP: time to peak
TURBT: transurethral resection of the bladder tumor
UCAs: ultrasound contrast agents
UCC: urothelial cell carcinoma
US: ultrasound
